# Norovirus transmission mediated by asymptomatic family members in households

**DOI:** 10.1371/journal.pone.0236502

**Published:** 2020-07-23

**Authors:** Benjarat Phattanawiboon, Nutthawan Nonthabenjawan, Patcharaporn Boonyos, Chanya Jetsukontorn, Worakarn Towayunanta, Kobkool Chuntrakool, Karn Ngaopravet, Kriangsak Ruchusatsawat, Ballang Uppapong, Somchai Sangkitporn, Eisuke Mekada, Yoshiharu Matsuura, Masashi Tatsumi, Hiroto Mizushima

**Affiliations:** 1 Thailand-Japan Research Collaboration Center on Emerging and Re-emerging Infections, Nonthaburi, Thailand; 2 Public Health Center 41, Bangkok, Thailand; 3 Department of Medical Sciences, National Institute of Health, Ministry of Public Health, Nonthaburi, Thailand; 4 Research and Education Promotion Foundation, Bangkok, Thailand; 5 Research Institute for Microbial Diseases, Osaka University, Osaka, Japan; Universita degli Studi di Parma, ITALY

## Abstract

The transmission of human norovirus excreted from infected persons occasionally causes sporadic infections and outbreaks. Both symptomatic patients and asymptomatic carriers have been reported to contribute to norovirus transmission, but little is known about the magnitude of the contribution of asymptomatic carriers. We carried out a 1-year survey of residents of a district of Bangkok, Thailand to determine the percentage of norovirus transmissions originating from asymptomatic individuals. We screened 38 individuals recruited from 16 families from May 2018 to April 2019 for GI and GII genotypes. Norovirus was detected every month, and 101 of 716 stool samples (14.1%) from individuals with no symptoms of acute gastroenteritis were norovirus-positive. The average infection frequency was 2.4 times per person per year. Fourteen genotypes were identified from the positive samples, with GII.4 being detected most frequently. Notably, 89.1% of the norovirus-positive samples were provided by individuals with no diarrhea episode. Similar to cases of symptomatic infections in Thailand, asymptomatic infections were observed most frequently in December. We detected 4 cases of NV infection caused by household transmission, and 3 of the 4 transmissions originated from asymptomatic individuals. We also identified a case in which norovirus derived from an asymptomatic individual caused diarrhea in a family member. These results suggest that asymptomatic individuals play a substantial role in both the maintenance and spreading of norovirus in a community through household transmission.

## Introduction

Human norovirus (NV) is a major causative agent of nonbacterial acute gastroenteritis (AGE). NV infections are observed throughout the year worldwide, and sporadic and pandemic infections of NV are a serious public health problem in both developing and developed countries [1–7]. NVs infect humans orally through direct or indirect contact with stool or vomit excreted from infected individuals [3, 8]. It has been suggested that contact with symptomatic individuals is a major route for NV transmission in a given community [9]. Outbreaks of NV often occur in semi-closed environments (e.g., nursing homes and hospitals) that facilitate viral transmission through person-to-person contacts [4, 8, 10]. Specific characteristics of NV, such as its low infectious dose and high shedding titer, have contributed to its maintenance in human communities [10, 11]. Because humans are a primary source of NV, it is assumed that NVs are retained in human communities throughout the cycle of infection and transmission.

Epidemiological studies have indicated that there are two NV-infected populations: one is AGE patients, and the other is individuals without diarrheal symptoms. The latter population, called “asymptomatic carriers” (which includes both pre- and post-disease-onset cases), is common worldwide [5, 12]. The ratio of asymptomatic NV infections ranges from <1% to >30%, depending on the setting [5, 12]. Asymptomatic NV infection occurs in all age groups, including children and infants [13–21]. A comprehensive analysis of the attack rates of NV during outbreaks suggested that asymptomatic infections occur in a proportion of the population [22]. More importantly, it has been reported that some outbreaks were traceable to NV transmission from asymptomatic food-handlers [23, 24].

Household contact is a risk factor for NV transmission [25, 26]. Living environments and household sizes have been proposed to affect the NV transmission rates in households [27]. Moreover, it has been reported that NVs may circulate in households via asymptomatic transmission [28]. However, little is known about the degree of the contribution of asymptomatic carriers to NV transmission among overall NV transmissions in a given community.

In this study, we carried out a survey of residents in a district of Bangkok, Thailand to identify the asymptomatic infection and transmission of NV. Our results showed that a variety of NV genotypes infected a proportion of the residents without inducing diarrheal symptoms. The number of asymptomatic infections of NV varied seasonally, as do symptomatic infections. We also discuss possible cases of household transmission from individuals asymptomatically infected with NV.

## Materials and methods

### Stool sample collection

This study was approved by the Bangkok Metropolitan Administration (Thailand) after being reviewed by the ethics committees of the Research Institute for Microbial Diseases, Osaka University (Japan, approval no. 29–11) and the Research and Education Promotion Foundation (Thailand, approval no. 2017–001). Stool samples were collected from 147 residents of Klong Toei District, Bangkok, Thailand. Klong Toei District, where Thailand’s largest slum is located, is composed of 41 distinct areas. Residents of all 41 areas were invited to participate in this study and consent was obtained from residents of 12 areas. Samples were collected in these 12 areas, which included 6 areas within slums and 6 more economically developed areas, and all 12 were geographically dispersed across Klong Toei District. A consent form for sample provision was obtained from all participants following an explanation by Public Health Center 41, a public medical institution in Bangkok. Consent of minors was obtained from their parent(s) or guardian(s).

Male or female residents who fulfilled all the following criteria were eligible for participation: (1) residency in the district for >1 year, (2) age 7–80 years, and (3) no symptoms of AGE (i.e., diarrhea, vomiting, abdominal pain, and fever) at the sample provision. Excretion of loose feces more than three times a day was defined as diarrhea in this study. The above inclusion criteria were applied to all surveys in this study. Samples were collected once/month for the primary survey at periods P1–P3, i.e., May 25, June 22, and July 20 2018. In the follow-up survey, samples were collected 1–3 times/month from July 11 2018 to April 11 2019 at periods F1–F16 (Table 1). A questionnaire survey on diarrhea episode(s) of participants and their families was conducted at every sample collection.

**Table 1:** The 1-year survey of 38 participants.

ID	Age	Family (size^3^)	Area No.	Type^4^	P1	P2	F1	P3	F2	F3	F4	F5	F6	F7	F8	F9	F10	F11	F12	F13	F14	F15	F16
					May 25	Jun 22	Jul 11	Jul 20	Jul 23	Jul 31	Aug 21	Sep 5	Sep 19	Oct 2	Oct 17	Nov 6	Dec 3	Dec17	Jan 8	Jan 24	Feb 15	Mar 11	Apr 11
**5**	**68**	**1 (1)**	**5**	**P**				**GI.3**				**NA**					**GII.4**						**NA**
**7**	**48**	**2 (1)**	**5**	**P**			**NA**	**NA**	**NA**	**NA**		**NA**	**NA**				**GII.4**		**NA**		**NA**	**NA**	**NA**
**11**	**50**	**3 (3)**	**7**	**S**			**GII.4**									***GII*.*4A***	***GII*.*4A***					**GII.4**	
**12**	**54**																						
**13**	**80**					**GII.4**	**GII.2**										***GII*.*4B***	***GII*.*4B***	**GI.3**				
**24**	**68**	**4 (15)**	**7**	**S**		**GII.4**			**NA**		**NA**							**NA**				**NA**	
**32**	**49**	**5 (4)**	**7**	**S**				**GII.2**^**1**^									**NA**			**NA**	**NA**	**NA**	
**48**	**36**	**6 (9)**	**3**	**P**						**GII.2**													
**49**	**13**																	**GII.4A**				**GI.3**	
**50**	**20**																**GII.4A**						
**51**	**47**																**GII.4A**						
**52**	**44**														**GI.7**		**GII.3**	**GII.4C**		**GII.3**		**GII.4**	
**53**	**39**						**NA**				**GI.7**				***GI*.*7***^***1***^	***GI*.*7***	**GII.8**	**GII.6**					
**54**	**41**						**NA**								***GI*.*7***	***GI*.*7***					**GII.4**		
**55**	**18**					**GII.4**										**GI.7**	**GII.4B**	**GII.6**	**GI.4, GII.8**				
**56**	**46**														**GI.7**			**GII.4A**		**GII.21**			
**57**	**24**	**7 (3)**	**3**	**P**		**GII.4**					**GI.3**						**GII.4A**	**GII.4B**		**GI.7**			
**58**	**28**							***GI*.*3***	***GI*.*3***								***GII*.*4A***	***GII*.*4A***	**GII.8**				
**59**	**28**								**GI.3**									**GII.4B**	**GI.4, GII.8**			**GII.8**	
**81**	**73**	**8 (4)**	**1**	**H**			**GII.13**										**GII.4**						
**81–1**	**48**										**GII.2**^2^				**GII.8**								
**81–2**	**16**									**GII.9**							**GII.4**						
**82**	**61**	**9 (1)**	**1**	**H**		**GII.4**							***GI*.*5***	***GI*.*5***			**GII.8**					**GII.4**	
**83**	**39**	**10 (1)**	**1**	**H**		**GII.4**		***GII*.*17***^2^	***GII*.*17***							**GI.3, GII.13**^**1**^	**GII.4**^**2**^						
**86**	**49**	**11 (2)**	**1**	**H**		**GII.4**			**NA**		**GI.5**^2^							**NA**			**NA**	**GII.4**	
**96**	**32**	**12 (2)**	**6**	**P**		**GI.7**^2^												**NA**					
**97**	**30**																	**NA**					
**101**	**26**	**13 (4)**	**6**	**P**		**GII.17**								**GII.4**			**GII.4**				**GII.4**		
**101m**	**58**																						
**101b**	**28**																**GII.8**						
**109**	**37**	**14 (5)**	**12**	**P**													**GII.4A**						
**110**	**44**				***GII*.*14***^***2***^	***GII*.*14***	***GII*.*14***	***GII*.*14***	***GII*.*14***	***GII*.*14***	***GII*.*14***	**GII.3**			**GII.2**								
**112**	**13**											**NA**		**NA**				**GII.8**^**1**^					**GII.2**
**113**	**11**					**GII.21**								**NA**	**NA**		**GII.8**	**GII.4B**			**GII.2**^**1**^		
**173**	**50**	**15 (1)**	**6**	**S**	**GII.17**					**NA**			**GII.4**			**NA**	**NA**	**NA**	**NA**		**NA**		
**181**	**53**	**16 (3)**	**9**	**H**													**GII.4**	**NA**					
**182**	**10**									**NA**		**NA**			**NA**		**GII.4**						**NA**
**183**	**9**					**GII.17**								**GII.4**	**NA**				**NA**				

Sample collection periods for the primary survey and the follow-up survey were indicated with P1 to P3 and F1 to F16, respectively.

Empty squares indicate NV-negative.

NA, not applicable because no sample was provided.

^1^Samples provided by individuals who developed diarrhea within 1 week before sample collection.

^2^Samples provided by individuals who developed diarrhea from 1 week to 1 month before sample collection.

^3^Household size.

^4^Area types: H, high-rise housing; P, public housing; S, slum

When members of the same family were infected with distinct genome sequences from GII.4 in the same or consecutive periods, genotypes are tentatively indicated as GII.4A, GII.4B or GII.4C.

Infections with the same or a closely similar strain lasting two or more consecutive periods in the same person were indicated with Italics.

Infections presumed to be involved in transmission are marked with gray.

### Detection of NVs by RT-PCR

For the detection of NVs by reverse transcription-polymerase chain reaction (RT-PCR), each 10% (w/v) stool suspension was prepared by suspension in phosphate buffered saline. RNA was extracted from each 10% stool suspension by using a Viral RNA Mini Kit (Qiagen, Hilden, Germany) according to the manufacturer's instruction. Semi-nested RT-PCR was performed to detect NV as described previously [29]: cDNA synthesis and the 1st PCR were done using the norovirus-specific primer sets COG1F and G1SKR (for GI) and COG2F and G2SKR (for GII) and the OneStep RT-PCR Kit (Qiagen). The 1st PCR product was used as the template to perform the 2nd PCR, using G1SKF and G1SKR (for GI) and G1SKF and G2SKR (for GII) and Ex Taq polymerase (Takara, Shiga, Japan). Amplified partial norovirus cDNA was detected by agarose gel electrophoresis.

### Determination of the NV genotypes and genogroups

The cDNA amplified by RT-PCR was purified from agarose gel using NucleoSpin Gel and PCR Clean-up (Macherey-Nagel, Düren, Germany). The purified cDNA was subjected to PCR reaction for sequencing by using BigDye terminator (Applied Biosystems, Carlsbad, CA). Nucleotide sequencing was done using a sequencer Model 3100 (Applied Biosystems). The genotype was determined by using Norovirus Typing Tool ver. 2.0 software [30].

### Measurement of the copy number

The copy number of the NV in each stool specimen was measured by real-time PCR as described previously with some modifications [31]. The norovirus genome was reverse-transcribed using SuperScript III reverse transcriptase (Thermo Fisher Scientific, Waltham, MA) and NV-specific primers. RING1-TPa and RING1-TPb were used as probes for GI genotypes and RING2-TP was used as a probe for GII genotypes. A plasmid encoding the NV genome was amplified with the use of COG1F and COG1R (for the GI genogroup) or COG2F and COG2R (for the GII genogroup) as a standard to calculate the copy number. When NV was not detected within 40 cycles of PCR, the copy number was considered undetectable. The copy number of each sample is expressed per gram of stool.

### Phylogenetic tree analysis

The evolutionary history was inferred by the neighbor-joining method [32]. The tree was drawn to scale, with the branch length in the same units as for the evolutionary distances used to infer the phylogenetic tree. We computed the evolutionary distances by using the maximum composite likelihood method; the distances are presented as the units of base substitutions per site. Evolutionary analysis was conducted by using MEGA7 software [33].

## Results

### Identification of asymptomatic NV infections

**Fig 1:**
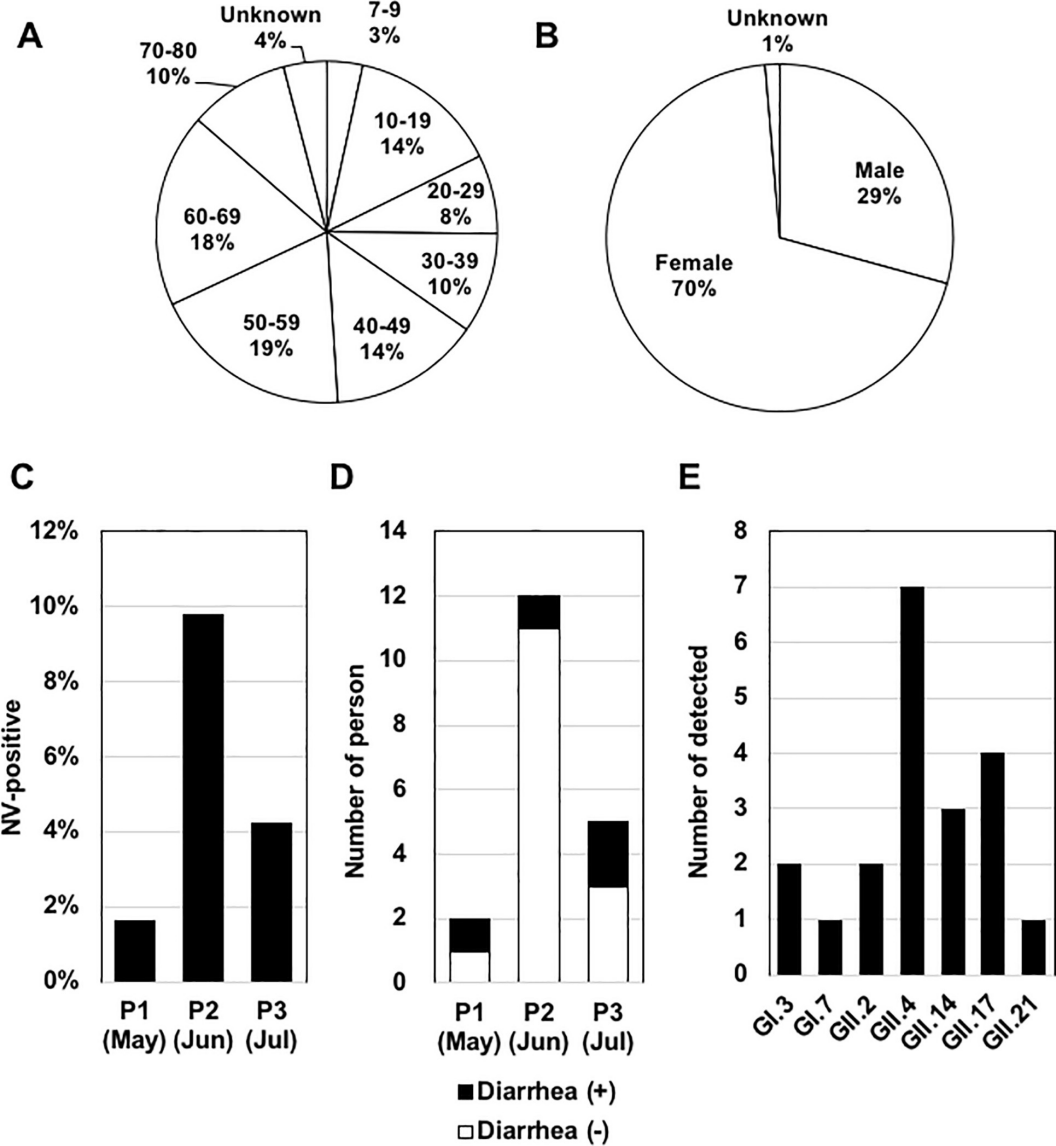
The primary survey of NV infections from May–July 2018. (A) Age distribution of the participants. (B) Gender distribution of the participants. (C) NV positivity in the primary survey. The numbers of NV-positive samples in each month were 2/128 (P1, collected at May 25), 12/123 (P2, collected at June 22), and 5/118 (P3, collected at July 20). (D) Diarrhea episodes among subjects providing NV-positive samples. The numbers of individuals with (*black bars*) and without (*white bars*) a diarrheal episode within 1 month prior to the sample collection among the NV-positive samples are shown. (E) The detection frequency of each genotype. The total number of samples with each genotype was counted.

To investigate asymptomatic NV infections, we initially collected stool samples from 147 residents of Bangkok's Klong Toei District. Samples were collected at approx. 1-month intervals from May to July 2018 (referred to as “the primary survey” hereafter), and 369 samples were obtained; the average recovery rate of samples was 84.2%. The distributions of age and gender of the participants are illustrated in Fig 1A and 1B. We excluded children <7 years old and adults >80 years old (who might need assistance from other family members to collect samples) to avoid an increase in the risk of NV transmission within each family.

NVs are divided into seven genogroups (GI to GVII), of which GI, GII, and GIV infect humans [1, 34, 35]. The majority of cases of NVs detected from AGE patients are classified into the GI and GII genogroups [36, 37], which can be subdivided into genotypes. Nine genotypes of GI and 22 genotypes of GII have been identified to date [1,34]. In the present study, the two major genogroups GI and GII were detected by RT-PCR. NV was detected from a proportion of the district's residents every month: P (primary) 1 (May), P2 (June), and P3 (July) (Fig 1C). Nineteen samples were identified as NV-positive.

The average positivity rate from May–July 2018 was 5.1% (19/369). Four of the 19 NV-positive samples were provided by individuals who had experienced diarrhea within 1 month before the sample collection, but the other 15 NV-positive samples were provided by individuals who had had no episode of diarrhea within 3 months before the sample collection (Fig 1D). Our sequence analysis of the 19 positive samples identified seven genotypes (GI.3, GI.7, GII.2, GII.4, GII.14, GII.17, and GII.21) (Fig 1E). GII.4 was detected in 7 samples and was the most common genotype. Co-infection of GI and GII was not detected. These results suggest that a certain number of individuals are asymptomatically infected with NV.

### Results of a 1-year survey of NV-positive individuals and their families

To obtain more details about asymptomatic NV infections in residents of the district, we carried out a follow-up survey of the individuals who were NV-positive in the primary survey from May–July 2018. In April 2018, a preliminary survey was conducted on 71 of the 147 participants. Participants ID7 (Family 2) and ID81 (Family 8) were positive for GI.5 and GII.2, respectively. Although these participants were NV-negative during the primary survey, they were recruited to the follow-up survey. Family members living with NV-positive individuals, regardless of relation, were also recruited to this survey whenever possible.

**Fig 2:**
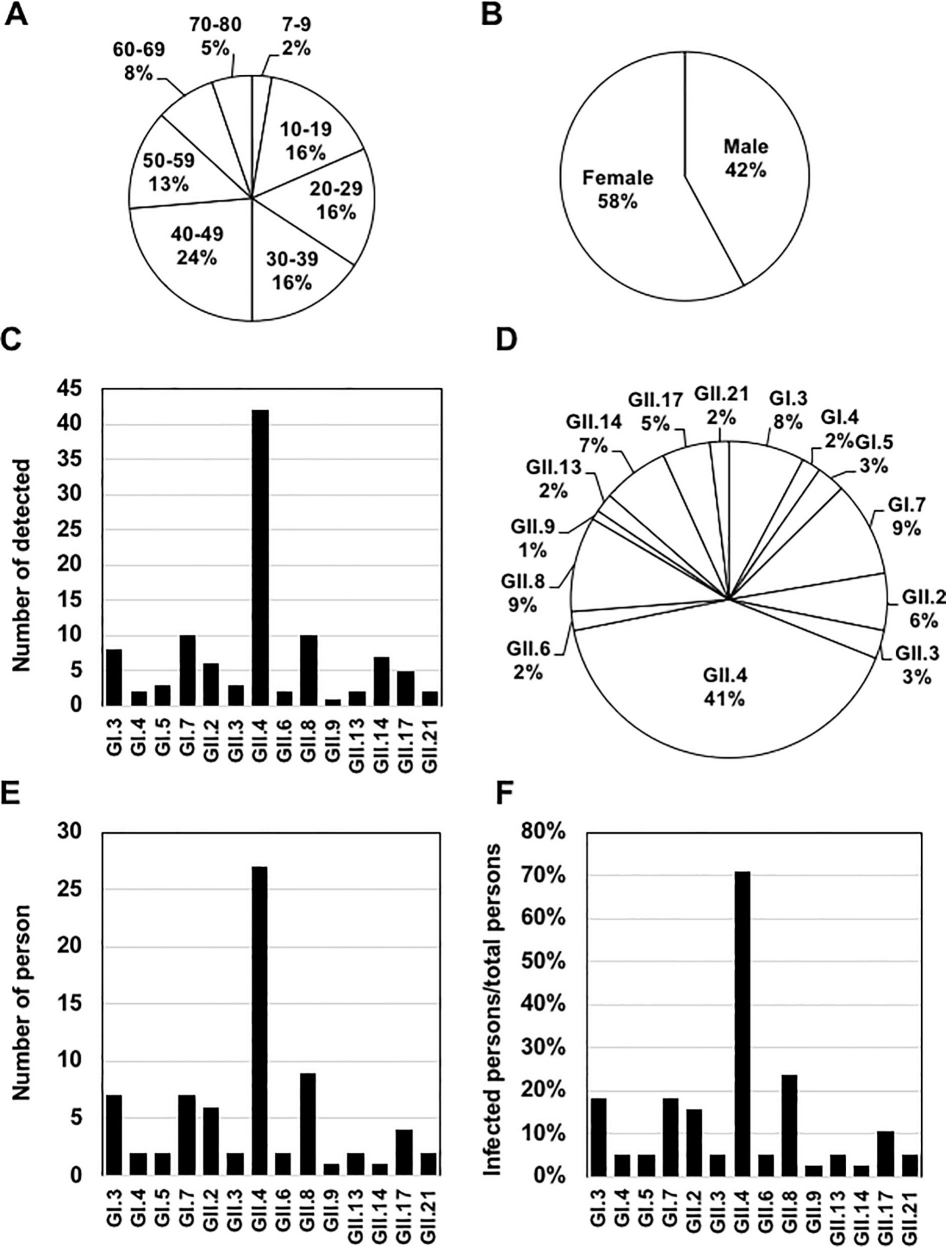
NV infections in the 1-year survey. (A) Age distribution of the participants. (B) Gender distribution of the participants. (C) The detection frequency of each genotype. The total number of samples with each genotype was counted. (D) The genotype distribution in the 1-year survey. (E) The number of participants infected with each genotype. (F) The ratio of participants infected with each genotype among the total participants.

NV-positive individuals were identified from 7 of the 12 areas during the preliminary and primary surveys. Thirty-eight members of 16 families (referred to as Families 1 to 16 hereafter) from the 7 areas participated in this follow-up survey (Table 1). All of the 38 members participated in this study from the primary survey (periods P1-P3). Their age and gender distributions are provided in Fig 2A and 2B. The sample collection periods, F (follow-up) 1 to F16, and dates are shown in Table 1. The follow-up survey was initiated on July 11 2018, with some overlap with the period of the primary survey, and continued until April 11 2019.

During a 1-year survey that combined the primary survey and follow-up survey, a total of 716 samples were collected and 101 samples (14.1%) were identified as NV-positive. Eleven of the 101 NV-positive samples were provided by individuals who had developed diarrhea within 1 month before the sample collection (see Table 1), and the remaining 90 samples (89.1%) were provided by individuals with no episode of diarrhea. Fourteen genotypes were identified from the positive samples: GI.1, GI.3, GI.5, GI.7, GII.2, GII.3, GII.4, GII.6, GII.8, GII.9, GII.13, GII.14, GII.17, and GII.21. GII.4 was detected the most frequently (Fig 2C), accounting for 41% of the positive samples (Fig 2D). GII.4 infection was detected in 27 individuals, which corresponds to 71.1% of the participants (Fig 2E and 2F).

**Fig 3:**
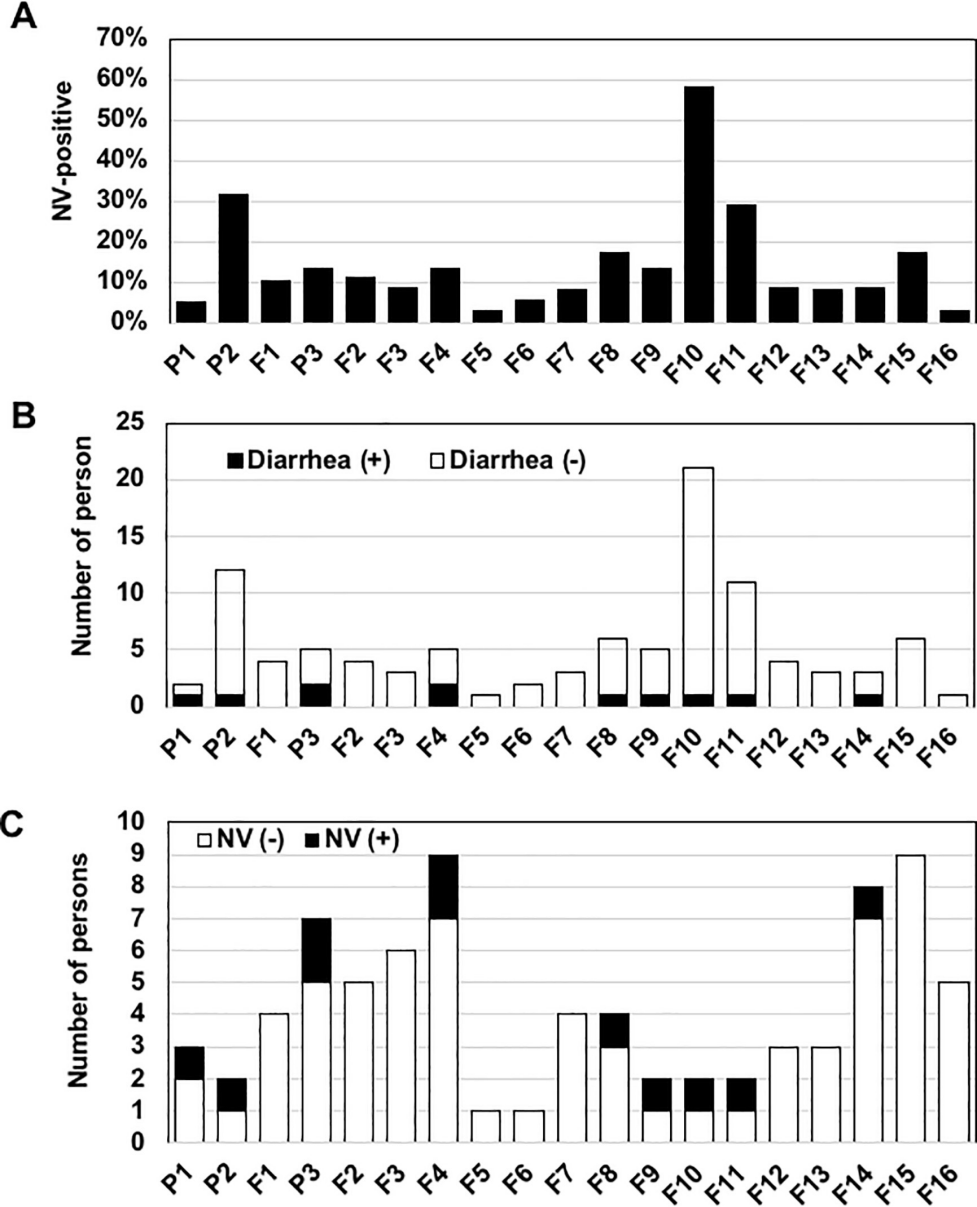
The monthly distribution of NV and individuals with diarrhea. (A) The monthly distribution of NV in the total samples. The NV positivity in samples collected each month is shown. (B) Diarrhea episodes of the NV-positive samples. The numbers of individuals with (*black bars*) or without (*white bars*) a diarrheal episode within 1 month prior to the sample collection are shown. (C) The monthly distribution of the incidence of diarrhea. The numbers of individuals who had an episode of diarrhea within 1 month prior to the sample collection among the NV-negative (*white bars*) and NV-positive individuals (*black bars*) in each month are shown.

NV was detected every month, and two peaks were observed in June and December (i.e., survey nos. P2 and F10) with 31.6% and 58.3% positivity, respectively (Fig 3A). However, no increase in diarrhea onset in NV-positive individuals was observed in these two months (Fig 3B). A certain proportion of participants developed diarrhea during the survey period, but the incidence of diarrhea was not significantly associated with NV infection (Fig 3C). These results suggest that (1) asymptomatic NV infection is a seasonal event, and (2) a relatively large population of NV is latent in humans without causing diarrheal symptoms.

### The excretion and copy numbers of NV

NV infection typically causes symptoms of AGE in immunocompetent hosts for 1 to 3 days [4, 38]. In humans who were experimentally inoculated with NV, shedding can be first detected 18–110 hr post-inoculation, and it lasts 13–56 days [38]. The duration of NV shedding was not significantly different between symptomatic patients and asymptomatic controls [39, 40]. Virus shedding lasts weeks to years when NV chronically infects immunocompromised hosts [41].

To estimate the duration of NV excretion among the participants in this survey, we analyzed the chronological changes in positivity and genotype for each individual. The data in Table 1 indicate that most of the NV-positive individuals excreted NV for approx. 1 month or less. There was no definitive difference in the duration of NV excretion between symptomatically and asymptomatically infected individuals. However, a unique case was identified (participant ID110), from whom GII.14 was excreted for nearly 3 months from May 2018 to August 2018. Our analysis of a partial NV genome sequence obtained from ID110 showed that it was the same NV strain that was excreted throughout the consecutive 3-month period (data not shown). Because ID110 developed diarrhea before weeks 1–4 of sample collection in May 2018, we suspect that ID110 had been persistently infected with the same strain of GII.14 since the disease onset.

The viral loads reported in asymptomatic individuals have differed among studies [42–45]. In the present study, the copy number of NV excreted in each sample was quantified by real-time RT-PCR in order to estimate the total copy number excreted from each participant. The copy numbers in selected samples varied widely among the individual samples, ranging from below the detection limit to >10^11^/g stool (Table 2). There was no clear correlation between the copy number of NV and the onset of diarrhea. In the case of long-term infection of GII.14 in participant ID110, the copy number was dynamically changed, ranging from below the detection limit to 1.62 × 10^7^/gram stool in each sample collection period. This result suggests that the viral copy number fluctuates during long-term infection.

**Table 2:** Copy number of NV detected in the 1-year survey.

ID	Age	Family	P1	P2	F1	P3	F2	F3	F4	F5	F6	F7	F8	F9	F10	F11	F12	F13	F14	F15	F16
**5**	**68**	**1**				3.44E+06				**NA**					ND						**NA**
**7**	**48**	**2**			**NA**	**NA**	**NA**	**NA**		**NA**	**NA**				UD		**NA**		**NA**	**NA**	**NA**
**11**	**50**	**3**			UD									UD	UD					ND	
**12**	**54**																				
**13**	**80**			UD	UD										UD	UD	UD				
**24**	**68**	**4**		5.44E+05			**NA**		**NA**							**NA**				**NA**	
**32**	**49**	**5**				3.94E+06									**NA**			**NA**	**NA**	**NA**	
**48**	**36**	**6**						UD													
**49**	**13**															ND				ND	
**50**	**20**														UD						
**51**	**47**														ND						
**52**	**44**												UD	1.07E+02	UD	ND		ND		ND	
**53**	**39**				**NA**				UD				UD	UD	ND*	3.30E+05					
**54**	**41**				**NA**								UD						ND		
**55**	**18**			UD										2.38E+06	ND*	UD	9.21E+05 (GI), 2.37E+06 (GII)				
**56**	**46**												UD			ND		ND			
**57**	**24**	**7**		UD					1.02E+05						8.06E+04	ND		ND			
**58**	**28**					2.68E+09	5.94E+07								UD	ND	1.95E+06				
**59**	**28**						8.30E+07									ND	5.7E+06 (GI), 5.16E+06 (GII)			ND	
**81**	**73**	**8**			UD										ND						
**81–1**	**48**								2.98E+05				UD							ND	
**81–2**	**16**							6.14E+06							UD						
**82**	**61**	**9**		8.10E+05							3.77E+04	UD			UD						
**83**	**39**	**10**		8.22E+05		8.53E+06	UD							1.17E+E11 (GI), UD (GII)	UD						
**86**	**49**	**11**		1.06E+06			**NA**		UD							**NA**					
**96**	**32**	**12**		1.37E+07												**NA**			NA		
**97**	**30**															**NA**					
**101**	**26**	**13**		UD								UD			1.25E+04				UD		
**101m**	**58**																				
**101b**	**28**														UD						
**109**	**37**	**14**													ND						
**110**	**44**		6.94E+06	7.66E+07	1.53E+05	1.62E+07	1.43E+05	UD	3.90E+06	UD			UD								
**112**	**13**									**NA**		**NA**				ND					ND
**113**	**11**			1.29E+06								**NA**	**NA**		ND	ND			ND		
**173**	**50**	**15**	1.33E+07					**NA**			UD			**NA**	**NA**	**NA**	**NA**		**NA**		
**181**	**53**	**16**													ND						
**182**	**10**							**NA**		**NA**			**NA**		ND						**NA**
**183**	**9**			UD								UD	**NA**				**NA**				

Empty squares indicate NV-negative.

Copy number values are shown per gram of stool.

NA, not applicable because no sample was provided.

UD, undetectable by real-time PCR.

ND, not determined.

ND*, not determined due to the sample being out-of-stock.

### The frequency of NV infection

**Fig 4:**
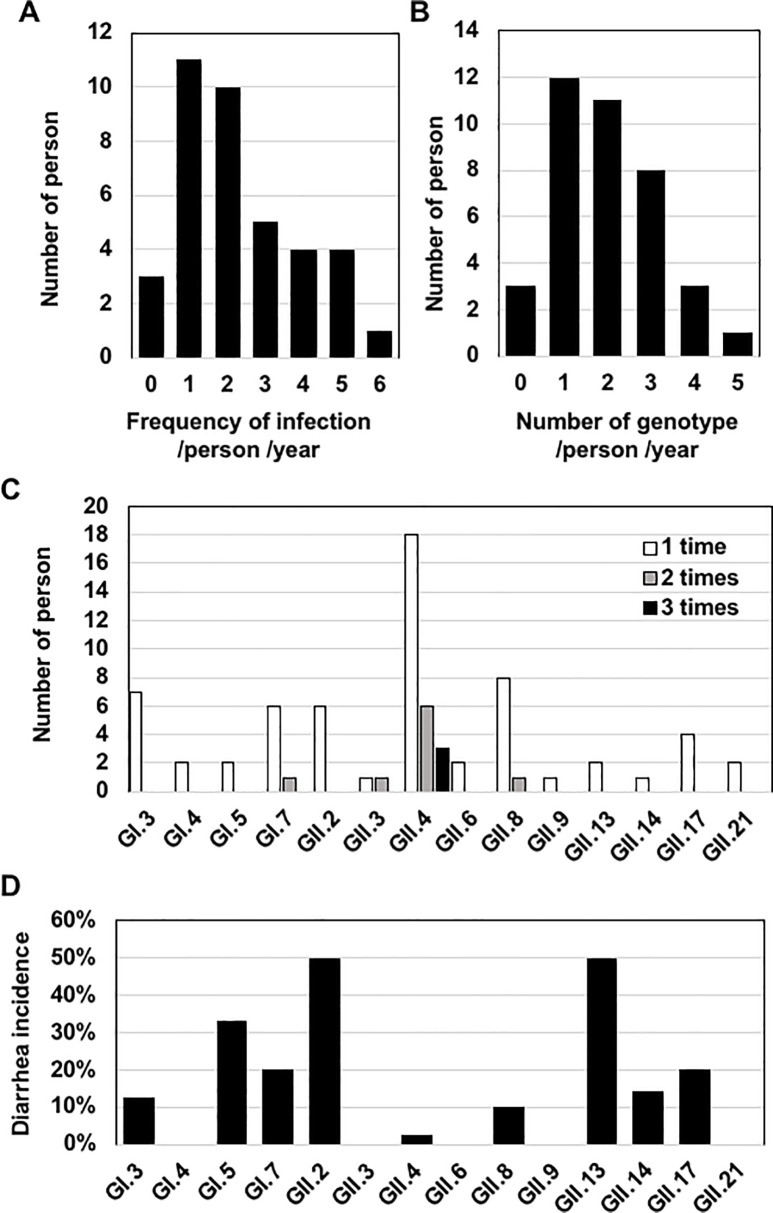
The NV infection frequency and the number of genotypes for each individual. **(**A) The frequency of NV infection. Infections of the same genotype that occurred more than 1-month interval were counted as 2 independent infections. When the same genotype was detected in consecutive periods, it was counted as one infection. Co-infection with 2 distinct genotypes during the same period was counted as 2 independent infections. The y-axis indicates the number of individuals who were infected with NV 0–5 times per year. (B) The number of genotypes detected for each individual. Infection was counted as described in panel A. The y-axis indicates the number of individuals who were infected with 0–4 genotypes per year. (C) The frequency of infection of each genotype in the same individual. Infection was counted as described in panel A. The y-axis indicates the number of individuals who were infected once 1 (*white bar*), 2 (*gray bar*), or 3 times (*black bar*) with each genotype. (D) The incidence of diarrhea of each genotype. The incidence of diarrhea is indicated by the percentage of samples associated with diarrhea among the total samples for each genotype.

We next analyzed the frequency of NV infection in each of the 38 individuals in the 16 families. Three participants (ID12, 97, and 101m) were not infected during the survey, but the other 35 participants (92%) were infected 1 or more times per year (Fig 4A). The average frequency of infection for the 38 participants was 2.4 times/year. When the number of genotypes in each individual was counted, we observed that 12 participants (31.6%) were infected with 1 genotype and 23 participants (60.5%) were infected with ≥2 genotypes (Fig 4B). Infections of 4 and 5 distinct genotypes were observed in 3 participants (ID59, 83, 113) and 1 participant (ID55), respectively. These results suggest that the frequencies of NV infection are highly divergent from person to person.

To gain insight into the relationship between the detection frequency and the infectivity of each genotype, we determined the number of infections for each genotype per person (Fig 4C). Ten genotypes infected 1 participant each, whereas GI.7, GII.3, GII.4, and GII.8 infected some of the participants 2–3 times at discontinuous periods. GI.7 infected participant ID53 in F4 and from F8 to F9 (Table 1). GII.3 infected participant ID52 in F10 and F13. GII.4 infected 6 participants (ID13, 52, 55, 82, 83, 86) 2 times and 3 participants (ID11, 57, 101) 3 times. GII.8 infected participant ID59 2 times in F12 and F15. The ability of each genotype to infect the same individual multiple times appeared to largely correlate with the detection frequency shown in Fig 2C.

To investigate the relationship between the detection frequency of each genotype and the disease incidence, we analyzed the incidence of diarrhea caused by infection with each genotype. No diarrhea incidence was observed from the individuals infected with GI.4, GII.3, GII.6, GII.9, or GII.21, and the remaining 9 genotypes showed various incidences of diarrhea (Fig 4D). Infection of the most common genotype (GII.4) showed a significantly low association with the incidence of diarrhea, and 41 of the 42 GII.4-positive samples were not associated with diarrhea onset. Together these results suggest that the detection frequency of each genotype does not correlate with the incidence of diarrhea.

### NV transmissions in households

To identify NV transmission in households, we analyzed the NV infections in 8 families from which multiple members participated (Families 3, 6, 7, 8, 12, 13, 14, and 16). Our analysis of NV-positive periods and genotypes for each family identified 7 possible cases of household transmission from Families 3, 6, 7, and 14 (Table 1).

**Fig 5:**
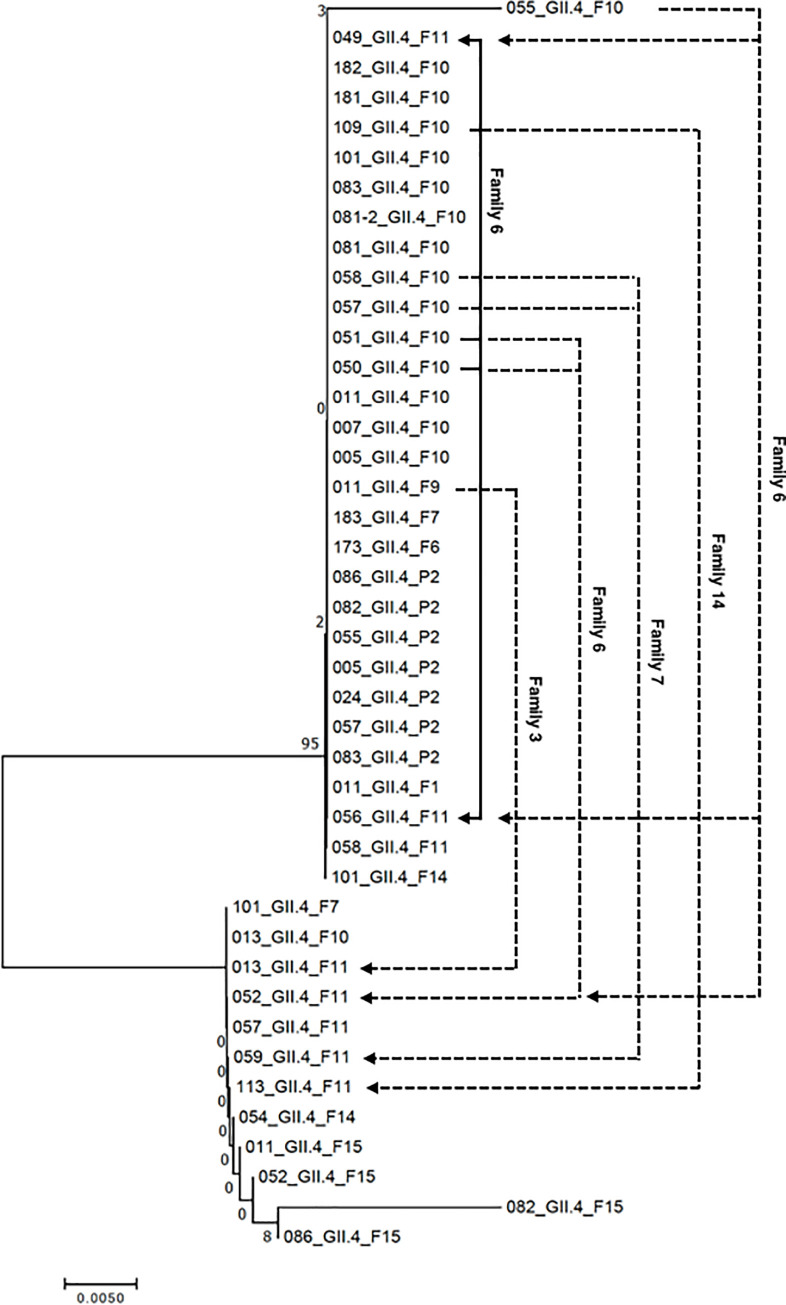
The phylogenetic tree of the GII.4 strains and possible transmission routes. The evolutionary history was inferred using the neighbor-joining method. The optimal tree with the sum of branch length = 0.06918009 is shown. The percentages of replicate trees in which the associated taxa clustered together in the bootstrap test (1,000 replicates) are shown next to the branches. The tree is drawn to scale, with branch lengths in the same units as those of the evolutionary distances used to infer the phylogenetic tree. Transmission routes with high probability (*solid arrows*) and low probability (*dashed arrows*) are indicated.

In Family 3, participant ID11 was GII.4-positive in F9, and participant ID13 became positive in the next period, F10. The phylogenetic tree analysis performed with the 282-base pair sequence of the open reading frame (ORF)2 5' region on the GII.4 strains detected in the 1-year survey revealed that ID11 and ID13 were independently infected with GII.4 strains classified into different clusters (Fig 5). Consistent with the phylogenetic tree analysis result, an 8-nucleotide difference was observed between these strains (S1 Fig).

In Family 6, 4 members (ID52, 53, 54, and 56) were GI.7-positive in F8, and another member (ID55) became GI.7-positive in the next period, F9. The comparison of genome sequences revealed that these family members were infected with the same strain (S2 Fig). Because ID53 developed diarrhea within 1 week before the sample collection in F8, GI.7 infecting ID53 appeared to be transmitted directly to ID55 or by secondary transmission through the 3 other family members (ID52, 54, and 56). In F10, this family had another infection of GII.4 in 3 members (ID50, 51, and 55), and another 3 members (ID49, 52 and 56) became GII.4-positive in the next period. The strains detected from ID50 and ID51 in period F10 showed high similarity to that from ID49 and ID56 in period F11, with differences at two nucleotide positions (Fig 5, S2 Fig), suggesting the occurrence of transmission. The differences in the genome sequence of strains observed from ID49, 50, 51, and 56 might occur during transmission processes. Other strains detected from ID55 and ID52 in periods F10 and F11, respectively, were classified into other clusters (Fig 5). These family members were thus infected with 3 strains, 1 of which was presumed to be involved in transmission, in periods F10 and F11.

Family 7 showed possible transmissions of GI.3 and GII.4 from P3 to period F2 and from period F10 to F11, respectively. Since the GI.3 strains detected from ID58 and ID59 in P3 and F2 were identical (S3 Fig), we suspect that the GI.3 was transmitted from ID58 to ID59. ID57 and ID58 were infected with the identical GII.4 strain in period F10, and this strain was detected only from ID58 in the next period (S3 Fig). ID57 and ID59 were infected with another GII.4 strain in F11 (Fig 5). Therefore, the infections of GII.4 that occurred during these periods were not cases of transmission.

In Family 14, participants ID109 and 113 were positive for GII.4 and GII.8, respectively in F10, and participants ID113 and 112 became positive for GII.4 and GII.8, respectively, in the next period. The GII.4 strain detected from ID109 in F10 was different from that of ID113 in F11, indicating that GII.4 infections in periods F10 and F11 occurred independently. The genome sequences of GII.8 detected from ID113 in period F10 and ID112 in F11 were identical. This suggested that GII.8 was transmitted from ID113 to ID112. Participant ID113 infected with GII.8 in F10 had no episode of diarrhea, but ID112 (who became GII.8-positive in F11) developed diarrhea.

Based on these results, we speculated that the 4 cases of infection with the same strains observed in consecutive periods in the same families were caused by household transmission. Almost all of the members of the families in which the transmissions occurred participated in this study: 9 of 9 members in Family 6; 3 of 3 in Family 7; and 4 of 5 in Family 14 (Table 1). Therefore, it is likely that the 4 transmissions occurred via the routes described above. Notably, 3 of the 4 cases of transmission originated from individuals with no diarrhea symptoms. In addition, 3 of the 4 cases of transmission were observed during Thailand's winter season. These results suggest that NV may be transmitted by asymptomatic individuals in households under the influence of unknown seasonal factor(s), and that NV derived from asymptomatic individuals can occasionally induce diarrhea in another individual.

## Discussion

### The latency of NV through asymptomatic infections and transmissions

We carried out a community-based survey to determine the extents of asymptomatic infection of NV and NV transmissions originating from asymptomatic individuals. Although it is well known that NV infection is associated with AGE, our present findings demonstrate that short-term sporadic infections of various genotypes of NV occur in a proportion of individuals through the year without inducing diarrheal symptoms. In the district studied in this survey, nearly 90% of the NV-positive samples analyzed in the 1-year survey were provided by individuals without a diarrhea episode. In addition, 4 possible cases of NV transmission originating from asymptomatic individuals were identified from multiple households, and 3 of the 4 possible transmission cases did not result in disease onset. These observations suggest that asymptomatic infection and transmission may be a major pathway that enables NV latency. The results of a previous study [28] also support this notion.

It has been reported that NV infection is associated with approx. 24%–50% of community-based cases of sporadic gastroenteritis [7, 46]. However, the transmission routes of NV for sporadic infections have not been verified, possibly due to the difficulty in tracing the sources of NV. In the present study, we observed that participant ID112, who was infected with NV derived from an asymptomatic individual (ID113) with no episode of AGE, developed diarrhea. This suggests that asymptomatic individuals in a community could be a source of NV causing sporadic infection associated with diarrheal symptoms.

This study determined whether each individual developed AGE before sample collection by examining episodes of diarrhea. However, since NV infections occasionally induce AGE without diarrhea [47], individuals who had AGE symptoms other than diarrhea might have been misclassified as asymptomatic. In addition, individuals with AGE symptoms at the sample collection were excluded from this study. These procedures might have resulted in overestimation of the contribution of asymptomatic individuals to the maintenance and transmission of NV. It is also undeniable that individuals who were prone to being asymptomatic might have been selected by selecting NV-positive individuals in the preliminary and primary surveys. However, our results suggest the necessity of further analyzing the role of asymptomatic individuals in a community. Since the frequency of asymptomatic infection varies depending on the setting [5, 12], analyses of individuals who are selected with an appropriate procedure with less bias are needed to verify the involvement of asymptomatic carriers in the latency and spreading of NV in the district and other settings.

### Possible mechanisms of asymptomatic infections

Our present findings and those of the prior study [28] indicate that a relatively high population of NV exists in a community by asymptomatic infection. Therefore, there should be mechanism(s) that enable NV to infect and propagate in humans without inducing diarrhea symptoms. GII.4 is the most prevalent genotype in AGE patients worldwide [4, 37, 48]. In Thailand, GII.4 has been prevalent in AGE patients for several decades [49, 50], and recent epidemiological studies carried out in Thailand by another group [51] and by our own group (unpublished data) showed that GII.4 is the most prevalent genotype in AGE patients. Nevertheless, our present data indicate that 41 of the 42 GII.4-positive samples in the 1-year survey were not associated with disease onset. About 70% of the participants were infected with GII.4 (Fig 2F), and 9 of the 38 participants were infected with GII.4 two or three times per year (Fig 4C). Our survey also showed that GI.7, GII.3 and GII.8 infected several individuals twice. These results suggest that a certain proportion of the residents examined may be vulnerable to asymptomatic infection for particular NV genotypes.

It has been shown that immunity to NV lasts ≥1 year post-infection [52]. Mucosal and cellular immune responses against NV in individuals who were pre-challenged with NV suppress the disease onset and reduce the viral load [53]. The Bangkok district examined in the present study includes slum areas, and households in some areas are settled on marshy areas where sewage is directly drained. The average household size in the slum is about 5 individuals, and the residences are densely situated. These characteristics of the residences might increase the frequency of the residents' NV exposure. Further research is necessary to explore the possibility that immunity acquired by exposure to prevalent genotypes, such as GII.4, permits asymptomatic infection and transmission.

Alternatively, the genetic backgrounds of humans and the genotypes of NV have been suggested to affect the severity of clinical outcomes [8, 52]. Symptomatic and asymptomatic individuals were reported to show differences in immune response and the alteration of intestinal flora upon NV infection [40, 54]. Since our present study was conducted for members of the same families — who have similar genetic backgrounds and share a common lifestyle and living environment that could affect intestinal flora — we cannot exclude the possibility that the frequent detection of asymptomatic infections was due to a bias arising from our participant selection. Further studies of greater numbers of participants are needed to examine the mechanism(s) underlying asymptomatic infections.

### The frequency of asymptomatic infection and transmission

Our findings demonstrate the possibility that asymptomatic individuals transmitted NV, resulting in symptomatic infection with diarrhea of other family members in their households. Since a short-term duration of NV shedding (<2 weeks) has often been observed for asymptomatic individuals [39, 40, 55, 56], the sample collection procedure that we used in the present study might not have covered all infections and transmissions. We observed cases in which multiple members of the same family were infected with viruses with the same genome sequence at the same period in addition to the cases of transmission. Two members of Family 7 (ID57, 58) and 2 members of Family 8 (ID81, 81–2) were infected with the same GII.4 strain in each family in period S10. The GII.8 detected from 2 members of Family 7 (ID58, 59) in period S12 was the identical strain (data not shown). For Family 6 in whom NV transmission was detected, very similar GII.4 strains were detected from 2 members (ID50, 51) in S10 prior to transmission.

These simultaneous infections with the same or a very similar strain in multiple family members might occur by transmission from one of the positive members, and transmission might occur more frequently than we estimated. It is necessary to collect samples at intervals of ≤1 week to clarify the precise process of NV transmission within families.

### The duration of NV infection in healthy individuals

Our results also demonstrate that NV shedding from asymptomatic individuals lasts approx. ≤1 month in most cases. A recent follow-up study showed that NV is excreted for 1–2 weeks from asymptomatic individuals [56]. The copy numbers of NV detected in our study were significantly higher compared to that study's data [56]. Since it has been shown that viral loads correlate with the duration of excretion from diarrhea patients [57, 58], the differences in viral loads between the previous and present study might be accounted for by the differences in the duration of shedding.

Long-term shedding of NV has been observed in immunocompromised patients [8,41]. We identified a case of long-term shedding of GII.14 that continued for nearly 3 months in participant ID110, who had no history of immunodeficiency during the survey. ID110 was also infected with GII.2 and GII.3, but long-term shedding was not observed for these genotypes. Long-term shedding might thus be a genotype-specific phenomenon. Since GII.14, a rare genotype in Thailand, is detected from <2% of AGE patients [59, 60], its pathogenicity and abilities of infection and transmission might be lower than those of the genotypes that are prevalent in AGE patients. This may be supported by our observation that no transmission of GII.14 to other family members of ID110 occurred.

### The seasonality of NV infection in Thailand

A seasonal infection of NV in symptomatic patients has been observed in many countries. A meta-analysis of global NV infection demonstrated apparent seasonality of NV infections in northern-hemisphere countries, with a peak in winter months (December to February) [6]. In Thailand, NV in AGE patients is detected mainly in the rainy season (June to October) and winter season (November to February) [61, 62]. Our present findings demonstrated that asymptomatic NV infection occurs seasonally, similar to symptomatic infection (Fig 3A), although there was no association of NV positivity with the incidence of disease (Fig 3B). A community-based epidemiological study showed that asymptomatic infections peak during the winter season in England [14], where seasonal symptomatic infections occur in the winter season [63]. Similar seasonal asymptomatic NV infections have been observed in Korea [64, 65] and Nicaragua [21]. As described above, symptomatic and asymptomatic infections also have similarities to the prevalent genotypes. Taking all of these findings together, we speculate that asymptomatic infections might be concurrent with symptomatic infections under the influence of unknown seasonal factor(s).

It has been reported that the distribution of genotypes detected from symptomatic children is quite different from that of asymptomatic children [21]. Simultaneous analyses of symptomatic and asymptomatic individuals of all age groups in the same settings may clarify the relationship between symptomatic and asymptomatic individuals regarding the prevalence of genotypes and their seasonal synchronicity.

In conclusion, our findings provide evidence suggesting a substantial role of asymptomatic individuals as a temporal reservoir mediating the propagation and transmission of NV in households. The present results indicate that a population of NVs was latent in a specific community by asymptomatic infection throughout a year. Further studies of the mechanisms of asymptomatic infection of NV capable of persistent infections and analyses designed to clarify the routes of NV transmission from/to asymptomatic carriers are necessary to prevent endemic NV infections and AGE caused by NV.

## Supporting information

S1 Fig Comparison of GII.4 genome sequences detected from Family 3 in periods F9 and F10. (PDF)

S2 Fig (A) Comparison of GI.7 genome sequences detected from Family 6 in periods F8 and F9. (B) Comparison of GII.4 genome sequences detected from Family 6 in periods S9 and S10.
(PDF)

S3 Fig (A) Comparison of GI.3 genome sequences detected from Family 7 in periods P3 and F2. (B) Comparison of GII.4 genome sequences detected from Family 7 in periods F10 and F11.
(PDF)

S4 Fig (A) Comparison of GII.4 genome sequences detected from Family 14 in periods F10 and F11. (B) Comparison of GII.8 genome sequences detected from Family 14 in periods F10 and F11.
(PDF)
