# Supplementary material for: Norovirus transmission mediated by asymptomatic family members in households
Source: PLoS One. 2020 Jul 23;15(7):e0236502. doi: 10.1371/journal.pone.0236502 (PMC7377487; doi:10.1371/journal.pone.0236502)
Supplement: S1 Fig — (PDF) [file pone.0236502.s001.pdf]

```
011_GII.4_S9  ATGAAGATGGCGTCGAGTGACGCCAACCCATCTGATGGGTCCGCAGCCAACCTCGTACCA 60
013_GII.4_S10 ATGAAGATGGCGTCGAGTGACGCCAACCCATCTGATGGGTCCGCAGCCAACCTCGTCCCA 60
              ******************************************************** ***

011_GII.4_S9  GAGGTCAACAATGAGGTTATGGCTTTGGAGCCCGTTGTTGGTGCCGCTATTGCGGCACCT 120
013_GII.4_S10 GAGGTCAACAATGAGGTTATGGCTCTGGAGCCCGTTGTTGGTGCCGCCATTGCGGCACCT 120
              ************************ ********************** ************

011_GII.4_S9  GTAGCGGGCCAACAAAATGTAATTGACCCCTGGATTAGAAATAATTTTGTACAAGCCCCT 180
013_GII.4_S10 GTAGCGGGCCAACAAAATGTAATTGACCCCTGGATTAGAAACAATTTTGTACAAGCCCCT 180
              ***************************************** ******************

011_GII.4_S9  GGTGGAGAGTTTACAGTGTCCCCTAGAAATGCTCCAGGTGAAATACTATGGAGCGCGCCT 240
013_GII.4_S10 GGTGGAGAGTTTACAGTATCCCCTAGAAACGCTCCAGGTGAAATACTATGGAGCGCGCCC 240
              ***************** *********** ***************************** 

011_GII.4_S9  CTGGGCCCTGACCTAAATCCCTATCTATCCCATTTGGCCAGA 282
013_GII.4_S10 TTGGGCCCTGATCTAAATCCCTACTTATCTCATTTGGCCAGA 282
               ********** ***********  **** ************
```

S1 Fig
