# Supplementary material for: Norovirus transmission mediated by asymptomatic family members in households
Source: PLoS One. 2020 Jul 23;15(7):e0236502. doi: 10.1371/journal.pone.0236502 (PMC7377487; doi:10.1371/journal.pone.0236502)
Supplement: S2 Fig — (A) Comparison of GI.7 genome sequences detected from Family 6 in periods F8 and F9. (B) Comparison of GII.4 genome sequences detected from Family 6 in periods S9 and S10. (PDF) [file pone.0236502.s002.pdf]

A

```
052_GI.7_S8 ATGATGATGGCGTCTAAGGACGCCCCTTCAAACATGGACGGCACTAGTGGTGCCGGTCAG 60
053_GI.7_S8 ATGATGATGGCGTCTAAGGACGCCCCTTCAAACATGGACGGCACTAGTGGTGCCGGTCAG 60
054_GI.7_S8 ATGATGATGGCGTCTAAGGACGCCCCTTCAAACATGGACGGCACTAGTGGTGCCGGTCAG 60
056_GI.7_S8 ATGATGATGGCGTCTAAGGACGCCCCTTCAAACATGGACGGCACTAGTGGTGCCGGTCAG 60
053_GI.7_S9 ATGATGATGGCGTCTAAGGACGCCCCTTCAAACATGGACGGCACTAGTGGTGCCGGTCAG 60
055_GI.7_S9 ATGATGATGGCGTCTAAGGACGCCCCTTCAAACATGGACGGCACTAGTGGTGCCGGTCAG 60
            ************************************************************

052_GI.7_S8 CTGGTACCAGAGGCAAACACAGCTGATCCTATCCCTTTGGAACCTGTGGTGGGAGCTGCA 120
053_GI.7_S8 CTGGTACCAGAGGCAAACACAGCTGATCCTATCCCTTTGGAACCTGTGGTGGGAGCTGCA 120
054_GI.7_S8 CTGGTACCAGAGGCAAACACAGCTGATCCTATCCCTTTGGAACCTGTGGTGGGAGCTGCA 120
056_GI.7_S8 CTGGTACCAGAGGCAAACACAGCTGATCCTATCCCTTTGGAACCTGTGGTGGGAGCTGCA 120
053_GI.7_S9 CTGGTACCAGAGGCAAACACAGCTGATCCTATCCCTTTGGAACCTGTGGTGGGAGCTGCA 120
055_GI.7_S9 CTGGTACCAGAGGCAAACACAGCTGATCCTATCCCTTTGGAACCTGTGGTGGGAGCTGCA 120
            ************************************************************

052_GI.7_S8 ACTGCAGCTGCTACAGCAGGCCAAGTTAATATGATTGACCCCTGGATTATGAATAATTTT 180
053_GI.7_S8 ACTGCAGCTGCTACAGCAGGCCAAGTTAATATGATTGACCCCTGGATTATGAATAATTTT 180
054_GI.7_S8 ACTGCAGCTGCTACAGCAGGCCAAGTTAATATGATTGACCCCTGGATTATGAATAATTTT 180
056_GI.7_S8 ACTGCAGCTGCTACAGCAGGCCAAGTTAATATGATTGACCCCTGGATTATGAATAATTTT 180
053_GI.7_S9 ACTGCAGCTGCTACAGCAGGCCAAGTTAATATGATTGACCCCTGGATTATGAATAATTTT 180
055_GI.7_S9 ACTGCAGCTGCTACAGCAGGCCAAGTTAATATGATTGACCCCTGGATTATGAATAATTTT 180
            ************************************************************

052_GI.7_S8 GTGCAATCGCCGGAAGGCGAGTTTACAATTTCCCCAAACAACACACCCGGTGACATCTTG 240
053_GI.7_S8 GTGCAATCGCCGGAAGGCGAGTTTACAATTTCCCCAAACAACACACCCGGTGACATCTTG 240
054_GI.7_S8 GTGCAATCGCCGGAAGGCGAGTTTACAATTTCCCCAAACAACACACCCGGTGACATCTTG 240
056_GI.7_S8 GTGCAATCGCCGGAAGGCGAGTTTACAATTTCCCCAAACAACACACCCGGTGACATCTTG 240
053_GI.7_S9 GTGCAATCGCCGGAAGGCGAGTTTACAATTTCCCCAAACAACACACCCGGTGACATCTTG 240
055_GI.7_S9 GTGCAATCGCCGGAAGGCGAGTTTACAATTTCCCCAAACAACACACCCGGTGACATCTTG 240
            ************************************************************

052_GI.7_S8 TTTGATCTGCAATTAGGACCCCATTTAAATCCATTTTTGCAACATCTCTCACAA 294
053_GI.7_S8 TTTGATCTGCAATTAGGACCCCATTTAAATCCATTTTTGCAACATCTCTCACAA 294
054_GI.7_S8 TTTGATCTGCAATTAGGACCCCATTTAAATCCATTTTTGCAACATCTCTCACAA 294
056_GI.7_S8 TTTGATCTGCAATTAGGACCCCATTTAAATCCATTTTTGCAACATCTCTCACAA 294
053_GI.7_S9 TTTGATCTGCAATTAGGACCCCATTTAAATCCATTTTTGCAACATCTCTCACAA 294
055_GI.7_S9 TTTGATCTGCAATTAGGACCCCATTTAAATCCATTTTTGCAACATCTCTCACAA 294
            ******************************************************
```

B

```
050_GII.4_S10 ATGAAGATGGCGTCGAGTGACGCCAACCCATCTGATGGGTCCGCAGCCAACCTCGTACCA 60
051_GII.4_S10 ATGAAGATGGCGTCGAGTGACGCCAACCCATCTGATGGGTCCGCAGCCAACCTCGTACCA 60
055_GII.4_S10 ATGAAGATGGCGTCGAGTGACGCCAACCCATCTGATGGGTCCGCAGCCAACCTCGTACCA 60
049_GII.4_S11 ATGAAGATGGCGTCGAGTGACGCCAACCCATCTGATGGGTCCGCAGCCAACCTCGTACCA 60
052_GII.4_S11 ATGAAGATGGCGTCGAGCGACGCCAACCCATCTGATGGGTCCGCAGCCAACCTCGTCCCA 60
056_GII.4_S11 ATGAAGATGGCGTCGAGTGACGCCAACCCATCTGATGGGTCCGCAGCCAACCTCGTACCA 60
              ***************** ************************************** ***

050_GII.4_S10 GAGGTCAACAATGAGGTTATGGCTTTGGAGCCCGTTGTTGGTGCCGCTATTGCGGCACCT 120
051_GII.4_S10 GAGGTCAACAATGAGGTTATGGCTTTGGAGCCCGTTGTTGGTGCCGCTATTGCGGCACCT 120
055_GII.4_S10 GAGGTCAACAATGAGGTTATGGCTTTGGAGCCCGTTGTTGGTGCCGCTATTGCGGCACCT 120
049_GII.4_S11 GAGGTCAACAACGAGGTTATGGCTTTGGAGCCCGTTGTTGGTGCCGCTATTGCGGCACCT 120
052_GII.4_S11 GAGGTCAACAATGAGGTTATGGCTCTGGAGCCCGTTGTTGGTGCCGCCATTGCGGCACCT 120
056_GII.4_S11 GAGGTCAACAACGAGGTTATGGCTTTGGAGCCCGTTGTTGGTGCCGCTATTGCGGCACCT 120
              *********** ************ ********************** ************

050_GII.4_S10 GTAGCGGGCCAACAAAATGTAATTGACCCCTGGATTAGAAATAATTTTGTACAAGCCCCT 180
051_GII.4_S10 GTAGCGGGCCAACAAAATGTAATTGACCCCTGGATTAGAAATAATTTTGTACAAGCCCCT 180
055_GII.4_S10 GTAGCGGGCCAACAAAATGTAATTAACCCCTGGATTAGAAATAATTTTGTACAAGCCCCT 180
049_GII.4_S11 GTAGCGGGCCAACAAAATGTAATTGACCCCTGGATTAGAAATAATTTTGTACAAGCCCCT 180
052_GII.4_S11 GTAGCGGGCCAACAAAATGTAATTGACCCCTGGATTAGAAACAATTTTGTACAAGCCCCT 180
056_GII.4_S11 GTAGCGGGCCAACAAAATGTAATTGACCCCTGGATTAGAAATAATTTTGTACAAGCCCCT 180
              ************************ **************** ******************

050_GII.4_S10 GGTGGAGAGTTTACAGTGTCCCCTAGAAATGCTCCAGGTGAAATACTATGGAGCGCGCCT 240
051_GII.4_S10 GGTGGAGAGTTTACAGTGTCCCCTAGAAATGCTCCAGGTGAAATACTATGGAGCGCGCCT 240
055_GII.4_S10 GGTGGAGAGTTTACAGTGTCCCCTAGAAATGCTCCAGGTGAAATACTATGGAGCGCGCCT 240
049_GII.4_S11 GGTGGAGAGTTTACAGTGTCCCCTAGAAATGCTCCAGGTGAAATACTATGGAGCGCGCCT 240
052_GII.4_S11 GGTGGAGAGTTTACAGTATCCCCTAGAAACGCTCCAGGTGAAATACTATGGAGCGCGCCC 240
056_GII.4_S11 GGTGGAGAGTTTACAGTGTCCCCTAGAAATGCTCCAGGTGAAATACTATGGAGCGCGCCT 240
              ***************** *********** *****************************

050_GII.4_S10 CTGGGACCTGACCTAAATCCCTATCTATCCCATTTGGCCAGA 282
051_GII.4_S10 CTGGGCCCTGACCTAAATCCCTATCTATCCCATTTGGCCAGA 282
055_GII.4_S10 CTGGGCCCTGACCTAAATCCCTATCTATCCCATTTGGCCAGA 282
049_GII.4_S11 CTGGGCCCTGACCTAAATCCCTATCTATCCCATTTGGCCAGA 282
052_GII.4_S11 TTGGGCCCTGATCTAAATCCCTACTTATCTCATTTGGCCAGA 282
056_GII.4_S11 CTGGGCCCTGACCTAAATCCCTATCTATCCCATTTGGCCAGA 282
               *** ***** *********** **** ************
```

S2 Fig
