# Supplementary material for: Norovirus transmission mediated by asymptomatic family members in households
Source: PLoS One. 2020 Jul 23;15(7):e0236502. doi: 10.1371/journal.pone.0236502 (PMC7377487; doi:10.1371/journal.pone.0236502)
Supplement: S3 Fig — (A) Comparison of GI.3 genome sequences detected from Family 7 in periods P3 and F2. (B) Comparison of GII.4 genome sequences detected from Family 7 in periods F10 and F11. (PDF) [file pone.0236502.s003.pdf]

A

```
058_GI.3_Jul ATGATGATGGCGTCTAAGGACGCCCCAACAAACATGGATGGCACTAGTGGTGCCGGCCAG 60
058_GI.3_S2  ATGATGATGGCGTCTAAGGACGCCCCAACAAACATGGATGGCACTAGTGGTGCCGGCCAG 60
059_GI.3_S2  ATGATGATGGCGTCTAAGGACGCCCCAACAAACATGGATGGCACTAGTGGTGCCGGCCAG 60
             ************************************************************

058_GI.3_Jul CTGGTACCAGAGGCAAATACAGCTGAGCCTATTGCTATGGATCCAGTAGTTGGTGCTGCC 120
058_GI.3_S2  CTGGTACCAGAGGCAAATACAGCTGAGCCTATTGCTATGGATCCAGTAGTTGGTGCTGCC 120
059_GI.3_S2  CTGGTACCAGAGGCAAATACAGCTGAGCCTATTGCTATGGATCCAGTAGTTGGTGCTGCC 120
             ************************************************************
058_GI.3_Jul ACGGCAGTTGCCACTGCTGGCCAAGTTAATATGATTGACCCCTGGATTATGAGCAATTTT 180
058_GI.3_S2  ACGGCAGTTGCCACTGCTGGCCAAGTTAATATGATTGACCCCTGGATTATGAGCAATTTT 180
059_GI.3_S2  ACGGCAGTTGCCACTGCTGGCCAAGTTAATATGATTGACCCCTGGATTATGAGCAATTTT 180
             ************************************************************

058_GI.3_Jul GTTCAAGCACCTCAAGGAGAGTTTACAATTTCACCCAATAATACACCTGGTGATATATTG 240
058_GI.3_S2  GTTCAAGCACCTCAAGGAGAGTTTACAATTTCACCCAATAATACACCTGGTGATATATTG 240
059_GI.3_S2  GTTCAAGCACCTCAAGGAGAGTTTACAATTTCACCCAATAATACACCTGGTGATATATTG 240
             ************************************************************

058_GI.3_Jul TTTGATCTACAATTGGGACCCCAATTAAACCCCTTTTTATCCCATTTGGCACAA 294
058_GI.3_S2  TTTGATCTACAATTGGGACCCCAATTAAACCCCTTTTTATCCCATTTGGCACAA 294
059_GI.3_S2  TTTGATCTACAATTGGGACCCCAATTAAACCCCTTTTTATCCCATTTGGCACAA 294
             ******************************************************
```

B

```
057_GII.4_S10 ATGAAGATGGCGTCGAGTGACGCCAACCCATCTGATGGGTCCGCAGCCAACCTCGTACCA 60
058_GII.4_S10 ATGAAGATGGCGTCGAGTGACGCCAACCCATCTGATGGGTCCGCAGCCAACCTCGTACCA 60
058_GII.4_S11 ATGAAGATGGCGTCGAGTGACGCCAACCCATCTGATGGGTCCGCAGCCAACCTCGTACCA 60
057_GII.4_S11 ATGAAGATGGCGTCGAGTGACGCCAACCCATCTGATGGGTCCGCAGCCAACCTCGTCCCA 60
059_GII.4_S11 ATGAAGATGGCGTCGAGTGACGTCAACCCATCTGATGGGTCCGCAGCCAACCTCGTCCCA 60
              ********************** ********************************* ***

057_GII.4_S10 GAGGTCAACAATGAGGTTATGGCTTTGGAGCCCGTTGTTGGTGCCGCTATTGCGGCACCT 120
058_GII.4_S10 GAGGTCAACAATGAGGTTATGGCTTTGGAGCCCGTTGTTGGTGCCGCTATTGCGGCACCT 120
058_GII.4_S11 GAGGTCAACAACGAGGTTATGGCTTTGGAGCCCGTTGTTGGTGCCGCTATTGCGGCACCT 120
057_GII.4_S11 GAGGTCAACAATGAGGTTATGGCTCTGGAGCCCGTTGTTGGTGCCGCCATTGCGGCACCT 120
059_GII.4_S11 GAGGTCAACAATGAGGTTATGGCTCTGGAGCCCGTTGTTGGTGCCGCCATTGCGGCACCT 120
              *********** ************ ********************** ************

057_GII.4_S10 GTAGCGGGCCAACAAAATGTAATTGACCCCTGGATTAGAAATAATTTTGTACAAGCCCCT 180
058_GII.4_S10 GTAGCGGGCCAACAAAATGTAATTGACCCCTGGATTAGAAATAATTTTGTACAAGCCCCT 180
058_GII.4_S11 GTAGCGGGCCAACAAAATGTAATTGACCCCTGGATTAGAAATAATTTTGTACAAGCCCCT 180
057_GII.4_S11 GTAGCGGGCCAACAAAATGTAATTGACCCCTGGATTAGAAACAATTTTGTACAAGCCCCT 180
059_GII.4_S11 GTAGCGGGCCAACAAAATGTAATTGACCCCTGGATTAGAAACAATTTTGTACAAGCCCCT 180
              ***************************************** ******************

057_GII.4_S10 GGTGGAGAGTTTACAGTGTCCCCTAGAAATGCTCCAGGTGAAATACTATGGAGCGCGCCT 240
058_GII.4_S10 GGTGGAGAGTTTACAGTGTCCCCTAGAAATGCTCCAGGTGAAATACTATGGAGCGCGCCT 240
058_GII.4_S11 GGTGGAGAGTTTACAGTGTCCCCTAGAAATGCTCCAGGTGAAATACTATGGAGCGCGCCT 240
057_GII.4_S11 GGTGGAGAGTTTACAGTATCCCCTAGAAACGCTCCAGGTGAAATACTATGGAGCGCGCCC 240
059_GII.4_S11 GGTGGAGAGTTTACAGTATCCCCTAGAAACGCTCCAGGTGAAATACTATGGAGCGCGCCC 240
              ***************** *********** ***************************** 

057_GII.4_S10 CTGGGCCCTGACCTAAATCCCTATCTATCCCATTTGGCCAGA 282
058_GII.4_S10 CTGGGCCCTGACCTAAATCCCTATCTATCCCATTTGGCCAGA 282
058_GII.4_S11 CTGGGCCCTGACCTAAATCCCTATCTATCCCATTTGGCCAGA 282
057_GII.4_S11 TTGGGCCCTGATCTAAATCCCTACTTATCTCATTTGGCCAGA 282
059_GII.4_S11 TTGGGCCCTGATCTAAATCCCTACTTGTCTCATTTGGCCAGA 282
               ********** ***********  * ** ************
```

S3 Fig
