# Supplementary material for: Norovirus transmission mediated by asymptomatic family members in households
Source: PLoS One. 2020 Jul 23;15(7):e0236502. doi: 10.1371/journal.pone.0236502 (PMC7377487; doi:10.1371/journal.pone.0236502)
Supplement: S4 Fig — (A) Comparison of GII.4 genome sequences detected from Family 14 in periods F10 and F11. (B) Comparison of GII.8 genome sequences detected from Family 14 in periods F10 and F11. (PDF) [file pone.0236502.s004.pdf]

A

```
109_GII.4_S10 ATGAAGATGGCGTCGAGTGACGCCAACCCATCTGATGGGTCCGCAGCCAACCTCGTACCA 60
113_GII.4_S11 ATGAAGATGGCGTCGAGTGACGCCAACCCATCTGATGGGTCCGCAGCCAACCTCGTCCCA 60
              ******************************************************** ***

109_GII.4_S10 GAGGTCAACAATGAGGTTATGGCTTTGGAGCCCGTTGTTGGTGCCGCTATTGCGGCACCT 120
113_GII.4_S11 GAGGTCAACAATGAGGTTATGGCTCTGGAGCCCGTTGTTGGTGCCGCCATTGCGGCACCT 120
              ************************ ********************** ************

109_GII.4_S10 GTAGCGGGCCAACAAAATGTAATTGACCCCTGGATTAGAAATAATTTTGTACAAGCCCCT 180
113_GII.4_S11 GTAGCGGGCCAACAAAATGTAATTGACCCCTGGATTAGAAACAATTTTGTACAAGCCCCT 180
              ***************************************** ******************

109_GII.4_S10 GGTGGAGAGTTTACAGTGTCCCCTAGAAATGCTCCAGGTGAAATACTATGGAGCGCGCCT 240
113_GII.4_S11 GGTGGAGAGTTTACAGTATCCCCTAGAAACGCTCCAGGTGAAATACTATGGAGCGCGCCC 240
              ***************** *********** ***************************** 

109_GII.4_S10 CTGGGCCCTGATCTAAATCCCTACCTATCCCATTTGGCCAGA 282
113_GII.4_S11 TTGGGCCCTGATCTAAATCCCTACTTATCTCATTTGGCCAGA 282
               *********************** **** ************
```

B

```
113_GII.8_S10 ATGAAGATGGCGTCGAATGACGCAGCTCCATCGAATGATGGCGCGGCTGGCCTCGTACCA 60
112_GII.8_S11 ATGAAGATGGCGTCGAATGACGCAGCTCCATCGAATGATGGCGCGGCTGGCCTCGTACCA 60
              ************************************************************

113_GII.8_S10 GAGATCAACCATGAGGTCATGGCCATAGAGCCTGTTGCAGGAGCCTCTTTAGCAGCTCCT 120
112_GII.8_S11 GAGATCAACCATGAGATCATGGCCATAGAGCCTGTTGCAGGAGCCTCTTTAGCAGCTCCT 120
              *************** ********************************************

113_GII.8_S10 GTCGTAGGACAGCTTAATATAATTGATCCCTGGATTAGAAATAATTTTGTGCAAGCCCCT 180
112_GII.8_S11 GTCGTAGGACAGCTTAATATAATTGATCCCTGGATTAGAAATAATTTTGTGCAAGCCCCT 180
              ************************************************************

113_GII.8_S10 GCTGGAGAATTCACTGTTTCGCCTAGGAATGCTCCAGGTGAATTTTTATTAGATCTAGAG 240
112_GII.8_S11 GCTGGAGAATTCACTGTTTCGCCTAGGAATGCTCCAGGTGAATTTTTATTAGATCTAGAG 240
              ************************************************************

113_GII.8_S10 TTAGGTCCAGAATTGAATCCCTATCTTGCTCACCTTGCACGC 282
112_GII.8_S11 TTAGGTCCAGAATTGAATCCCTATCTTGCTCACCTTGCACGC 282
              ******************************************
```

S4 Fig
